# Antibody preparation and age-dependent distribution of TLR8 in Bactrian camel spleens

**DOI:** 10.1186/s12917-023-03812-z

**Published:** 2023-12-16

**Authors:** Ying-Dong Fang, Jing-Yu Liu, Fei Xie, Li-Ping Liu, Wei-Wei Zeng, Wen-Hui Wang

**Affiliations:** College of Veterinary Medicine, Gansu Agricultural University, Lanzhou, 730070 China

**Keywords:** Bactrian camels, Toll-like receptor 8, Antibody preparation, Spleen, Aging, Distribution

## Abstract

**Background:**

Toll-like receptor 8 (TLR8) can recognize specific pathogen-associated molecular patterns and exert multiple immunological functions through activation of signaling cascades. However, the precise distribution and age-related alterations of TLR8 in the spleens of Bactrian camels have not yet been investigated. This study aimed to prepare a rabbit anti-Bactrian camel TLR8 polyclonal antibody and elucidate the distribution of TLR8 in the spleens of Bactrian camels at different age groups. The methodology involved the construction of the pET-28a-TLR8 recombinant plasmid, followed by the expression of TLR8 recombinant protein via prokaryotic expression. Subsequently, rabbits were immunized with the purified protein to prepare the TLR8 polyclonal antibody. Finally, twelve Alashan Bactrian camels were categorized into four groups: young (1–2 years), pubertal (3–5 years), middle-aged (6–16 years) and old (17–20 years). These camels received intravenous sodium pentobarbital (20 mg/kg) anesthesia and were exsanguinated to collect spleen samples. Immunohistochemical techniques were employed to observe and analyze the distribution patterns and age-related changes of TLR8 in the spleen.

**Results:**

The results showed that the TLR8 recombinant protein was expressed in the form of inclusion body with a molecular weight of 52 kDa, and the optimal induction condition involved 0.3 mmol/L IPTG induction for 8 h. The prepared antibody yielded a titer of 1:32 000, and the antibody demonstrated specific binding to TLR8 recombinant protein. TLR8 positive cells exhibited a consistent distribution pattern in the spleen across different age groups of Bactrian camels, primarily scattered within the periarterial lymphatic sheath of the white pulp, marginal zone, and red pulp. The predominant cell type expressing TLR8 was macrophages, with expression also observed in neutrophils and dendritic cells. Statistical analysis revealed that there were significant differences in the distribution density of TLR8 positive cells among different spleen regions at the same age, with the red pulp, marginal zone, and white pulp showing a descending order (*P*<0.05). Age-related changes indicated that the distribution density in the marginal zone and red pulp exhibited a similar trend of initially increasing and subsequently decreasing from young to old camels. As camels age, there was a significant decrease in the distribution density across all spleen regions (*P*<0.05).

**Conclusions:**

The results confirmed that this study successfully prepared a rabbit anti-Bactrian camel TLR8 polyclonal antibody with good specificity. TLR8 positive cells were predominantly located in the red pulp and marginal zone of the spleen, signifying their pivotal role in the innate immune response of the spleen. Aging was found to significantly reduce the density of TLR8 positive cells, while leaving their scattered distribution characteristics unaffected. These findings provide valuable support for further investigations into the immunomorphology and immunosenescence of the spleen in Bactrian camels.

**Supplementary Information:**

The online version contains supplementary material available at 10.1186/s12917-023-03812-z.

## Background

Innate immunity serves as the body’s first line of defense against invading pathogenic microorganisms, and the mammalian innate immune system detects pathogenic microorganisms invading the body through pattern recognition receptors encoded by germline genes [1, 2]. Among these receptors, Toll-like receptors (TLRs) play a pivotal role in initiating innate immune responses by recognizing pathogen-associated molecular patterns (PAMPs), which are conserved molecular structures found in pathogens, and damage-associated molecular patterns (DAMPs), components released by damaged cells and tissues [2, 3]. Activation of TLRs can induce multiple biological effects, such as inflammatory responses, modulation of cell cycle, apoptosis, and regulation of cell metabolism [4]. TLRs, as a “bridge” connecting innate immunity and adaptive immunity, are classified into two categories based on their subcellular localization. The first category comprises TLRs located on the plasma membrane in the form of heterodimers or homodimers, including TLR1, TLR2, TLR4, TLR5, TLR6, and TLR10, primarily recognizing bacterial cell wall components and related molecules. The second category includes TLRs expressed on endosomes and phagosome membranes in the form of homodimers, such as TLR3, TLR7, TLR8, and TLR9, which predominantly recognize nucleic acids from bacteria and viruses [5, 6].

TLR8, a prominent member of the TLR7/8/9 subfamily, is a type I membrane-spanning proteins. It consists of an extracellular leucine-rich repeat (LRR) domain responsible for ligand recognition, a transmembrane domain, and an intracellular Toll/IL-1 receptor (TIR) domain responsible for signal transduction [7, 8]. TLR8 is primarily expressed in monocytes/ macrophages, neutrophils, dendritic cells, and natural killer cells [9, 10]. It has the capacity to recognize single-stranded RNA [9, 11], synthetic oligonucleotides [10], guanosine analogs with antiviral activities [11], synthetic imidazoquinoline compounds [12], and activate specific signaling pathways to exert multiple immunological functions.  TLR8 recognizes its corresponding PAMPs and, upon binding with PAMP, brings the intracellular TIR domains into close proximity to each other, thereby initiating signal transduction. The TIR domains can bind to the adapter protein myeloid differentiation factor 88 (MyD88) through homotypic interaction, which subsequently recruits serine-threonine kinases IL-1R-associated kinase 1 (IRAK1) and IRAK4, and activates the ubiquitin ligase TNF receptor-associated factor 6 (TRAF6). TRAF6 can activate downstream transcription factors, such as NF-κB and interferon regulatory factor 7 (IRF7), by initiating the corresponding signal transduction pathways. Ultimately, pro-inflammatory cytokines, such as TNF-α, IL-1β, IL-6, IL-12, IL-27, and type I interferon are produced to participate in the body's innate and adaptive immune response [7, 13, 14]. Activation of the TLR8 signaling pathway leads to the production of  IFN-α and pro-inflammatory cytokines such as TNF-α, IL-1β, IL-6, IL-12, suggesting that TLR8 plays an important role in cellular immunity, anti-tumor, antiviral and anti-infection of the body. Furthermore, Th2-type cytokines, such as IL-4, IL-5 and IL-13, are associated with the occurrence and development of certain allergic diseases. TLR8 can exert anti-allergic effects by inhibiting the aberrant secretion of Th2-type cytokines [15]. Notably, CD4^+^regulatory T (Treg) cells can suppress the body's immune responses, while TLR8 can activate the TLR8-MyD88-IRAK4 signaling pathway through recognition of ligands to inhibit the immunosuppressive function of Treg cells, thereby enhancing the body's anti-tumour immunity [16]. Therefore, basic studies on the distribution, expression and signaling mechanism of TLR8 are of great significance for revealing the relevant mechanisms of the body's anti-infection, anti-tumor and anti-allergy.

As of now, research on TLR8 has been reported in humans [17, 18] and many animals, mainly including bovine [19], goat [20], porcine [21], equine [22],* nyctereutes procyonoides* [23], mouse [24, 25] and other animals [26–28]. These studies are mainly concerned with the molecular cloning, characterization and expression patterns of TLR8 [22–28], the development and clinical application of TLR8 agonists [17, 29], particularly in the context of tumor immunotherapy [17], the intricacies of the TLR8 signaling pathway and its role in anti-infection mechanisms [19, 21, 26]. Furthermore, the correlation between TLR8 and the occurrence and development of some clinical diseases has also been reported, such as cancer [17], tuberculosis [18] and asthma [29]. The Bactrian camel, an economically important livestock species in northwest China, is a special species because of its ability to survive in arid and semi-arid desert environments [30]. Distinguishing itself from other ruminants, Bactrian camels possess distinctive biological characteristics, such as greater resistance to rough-feeding, hunger, thirst, heat, cold and so on [31, 32]. Furthermore, studies on the mucosal immunity of Bactrian camels have also revealed that they have distinctive mucosal immunological characteristics [33, 34]. The spleen, as the largest immune organ in Bactrian camels, is a key site for lymphocyte migration and settlement, as well as immune response. It also serves essential biological functions, such as filtering and storing blood, regulating blood volume, and participating in the body's immune response [35]. As a highly expressed site of TLR8, the spleen can generate corresponding immune responses when the body is stimulated by antigens. The diverse biological effects mediated by TLR8 may be an essential component in maintaining the strong immune function of Bactrian camels. Despite the comprehensive understanding of TLR8 distribution and expression patterns in many animals, there is a noticeable gap in research concerning TLR8 in Bactrian camels. Specifically, the distribution characteristics and age-related changes of TLR8 in the spleens of Bactrian camels remain unexplored. As an important reagent necessary for basic research, polyclonal antibodies are widely used due to their short preparation cycle and high cost-effectiveness. However, there are still no commercial Bactrian camel TLR8 antibodies. Consequently, the aim of this study was to obtain the TLR8 recombinant protein of Bactrian camel through prokaryotic expression, and prepare the rabbit anti-Bactrian camel TLR8 polyclonal antibody. Also to observe, analyze and compare the distribution characteristics and age-related changes of TLR8 in the spleens of Bactrian camels as a support for further studies on the immunomorphology and immunosenescence of Bactrian camel spleens and mechanism of TLR8 signaling pathway.

## Results

### TLR8 gene synthesis and recombinant plasmid construction of Bactrian camel

**Fig. 1:**
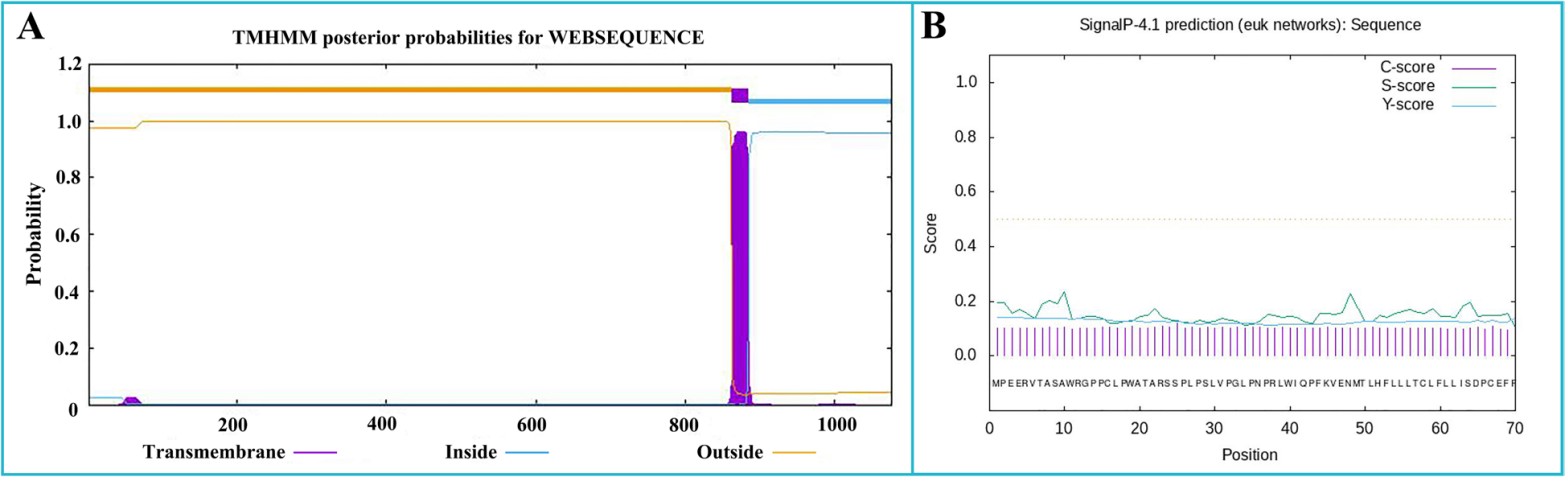
Prediction of the transmembrane region and signal peptide of the TLR8 protein in Bactrian camels. **A **Prediction of the transmembrane region of the TLR8 protein in Bactrian camels; **B** Prediction of the signal peptide of the TLR8 protein in Bactrian camels

**Fig. 2:**
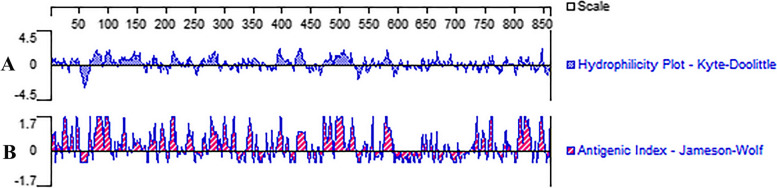
The hydrophilicity and antigenic epitope of the TLR8 protein in Bactrian camels. **A** The hydrophilicity of the TLR8 protein in Bactrian camels;** B** The antigenic epitope of the TLR8 protein in Bactrian camels

**Fig. 3:**
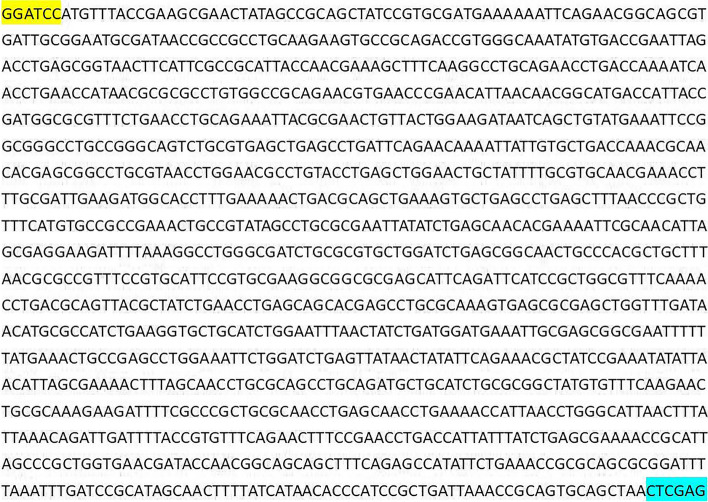
Optimized sequence of TLR8 gene in Bactrian camels. 5’ add *Bam*H I (Yellow) and 3’ add *Xho *I (Cyan)

**Fig. 4:**
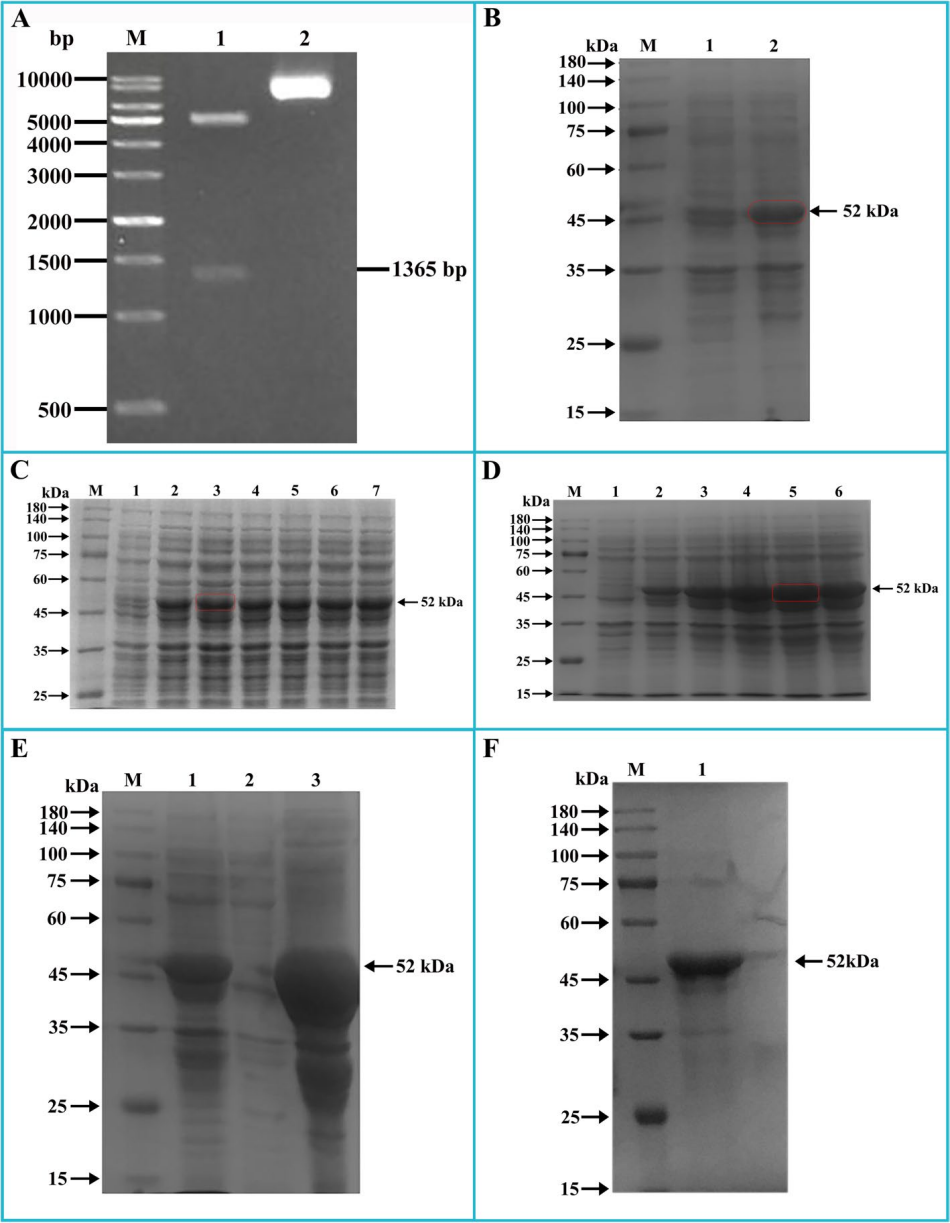
Prokaryotic expression of Bactrian camel TLR8.** A **Double enzyme digestion results of the TLR8 recombinant plasmid; **M** DL10000 DNA Marker;** 1 **pET-28a-TLR8 was digested with* BamH* I and *Xho* I; **2** pET-28a (+).** B** Induced expression results of the TLR8 recombinant plasmid; **M** Protein molecular weight Marker. The same below; **1** Pre-induction products of recombinant bacteria; **2** Recombinant bacteria induced products. **C **Optimization of IPTG concentration of the TLR8 recombinant protein; **1** Pre-induction products of recombinant bacteria; ** 2-7** Recombinant bacteria were induced by IPTG concentration of 0.1 mmol/L, 0.3 mmol/L, 0.5 mmol/L, 0.7 mmol/L, 0.9 mmol/L and 1.0 mmol/L. **D **Optimization of expression time of the TLR8 recombinant protein; **1** Pre-induction products of recombinant bacteria; **2-6** Products of recombinant bacteria induced for 2 h, 4 h, 6 h, 8 h, and 10 h. **E **Expression form of the TLR8 recombinant protein; **1** Before ultrasonication of the recombinant bacteria after induction; **2** Lysis supernatant of the recombinant bacteria after induction; **3** Lysis precipitate of the recombinant bacteria after induction. **F** Purification of the TLR8 recombinant protein; **1** Purified recombinant protein

The prediction of the transmembrane structure (Fig. 1A) showed that the Bactrian camel TLR8 protein consisted of a total of 1078 amino acids, with 1-862 amino acids constituting the extra-membrane region. This extra-membrane region was intercepted using the Editseq program of DNAStar7.0. Additionally, the prediction of the signal peptide (Fig. 1B) indicated the absence of a signal peptide in the TLR8 protein. Analysis of protein hydrophilicity (Fig. 2A) and antigenic epitope prediction (Fig. 2B) revealed that the Bactrian camel TLR8 protein was hydrophobic and possessed a high antigenic index. The extra-membrane amino acid sequence (70-518 amino acids) with a high antigenic index was intercepted using the Editseq program of DNAStar7.0. Upstream of the corresponding base sequence, the* BamH* I restriction site and the start codon were appended, while downstream, the *Xho* I restriction site and the stop codon were added. The optimized sequence of the Bactrian camel TLR8 gene was 1365 bp in length (Fig. 3), encoding a total of 450 amino acids, with a molecular weight of 52 kDa. Double digestion identification of the recombinant plasmid confirmed the presence of a distinct band between 1 000 and 1 500 bp on the DL10000 DNA Marker, consistent with expectations, thus confirming the successful construction of the recombinant plasmid pET-28a-TLR8 (Fig. 4A).

### Prokaryotic expression results of pET-28a-TLR8 recombinant plasmid

The recombinant bacteria were cultured at 37°C until the OD_600_ value reached 0.8, at which point the bacteria were induced with 1.0 mmol/L IPTG at 37°C for 6 h. The pre-induction and induction products of the recombinant bacteria were subjected to 12% SDS-PAGE detection. Results showed that following IPTG induction, a clear band appeared between 45 and 60 kDa (Fig. 4B), with a size of 52 kDa, when compared to the pre-induction products. This indicated that the recombinant plasmid pET-28a-TLR8 had been successfully transferred into competent cells BL21 and expressed normally.

### Optimization results of expression conditions of TLR8 recombinant protein in Bactrian camel

The recombinant bacteria were induced to express TLR8 protein with different concentrations of IPTG (0.1-1.0 mmol/L). The results of 12% SDS-PAGE revealed that the highest expression level of the target protein occurred at an IPTG concentration of 0.3 mmol/L (Fig. 4C). Therefore, 0.3 mmol/L was determined as the optimal IPTG induction concentration for TLR8 recombinant protein expression.

Under the optimal IPTG concentration, the recombinant bacteria were induced to express TLR8 protein at different time gradients (2-10 h). The results of 12% SDS-PAGE indicated (Fig. 4D) that the target protein was expressed when IPTG was added for 2 h. As the induction time passed, the expression level of the target protein gradually increased, reached the highest level at 8 h, and then gradually decreased. Therefore, the optimal induction time for TLR8 recombinant protein expression was at 8 h.

### Purification results of TLR8 recombinant protein in Bactrian camel

Under the optimal induction conditions, following induction and disruption of the recombinant bacteria, the lysed supernatant and precipitate were collected after centrifugation. Analysis of 12% SDS-PAGE results showed (Fig. 4E) that a clear target band, with a size of 52 kDa, was exclusively present in the precipitate of the recombinant bacteria. This indicated that the TLR8 recombinant protein was successfully expressed in the form of inclusion bodies in competent cells BL21.

Subsequently, the recombinant bacteria were induced and cultured, then disrupted by ultrasound and centrifuged to collect the lysed precipitate for protein purification. The eluate was collected during protein purification, and the highest protein content determined by Ultraviolet spectrophotometer was 2.009 mg/mL. The results of 12% SDS-PAGE demonstrated a single band without other heteroproteins, confirming the successful purification of the target protein, with a size of 52 kDa, suitable for use in the immunization of experimental animals (Fig. 4F).

### Serum titer and specificity detection of rabbit anti-Bactrian camel TLR8 polyclonal antibody

**Fig. 5:**
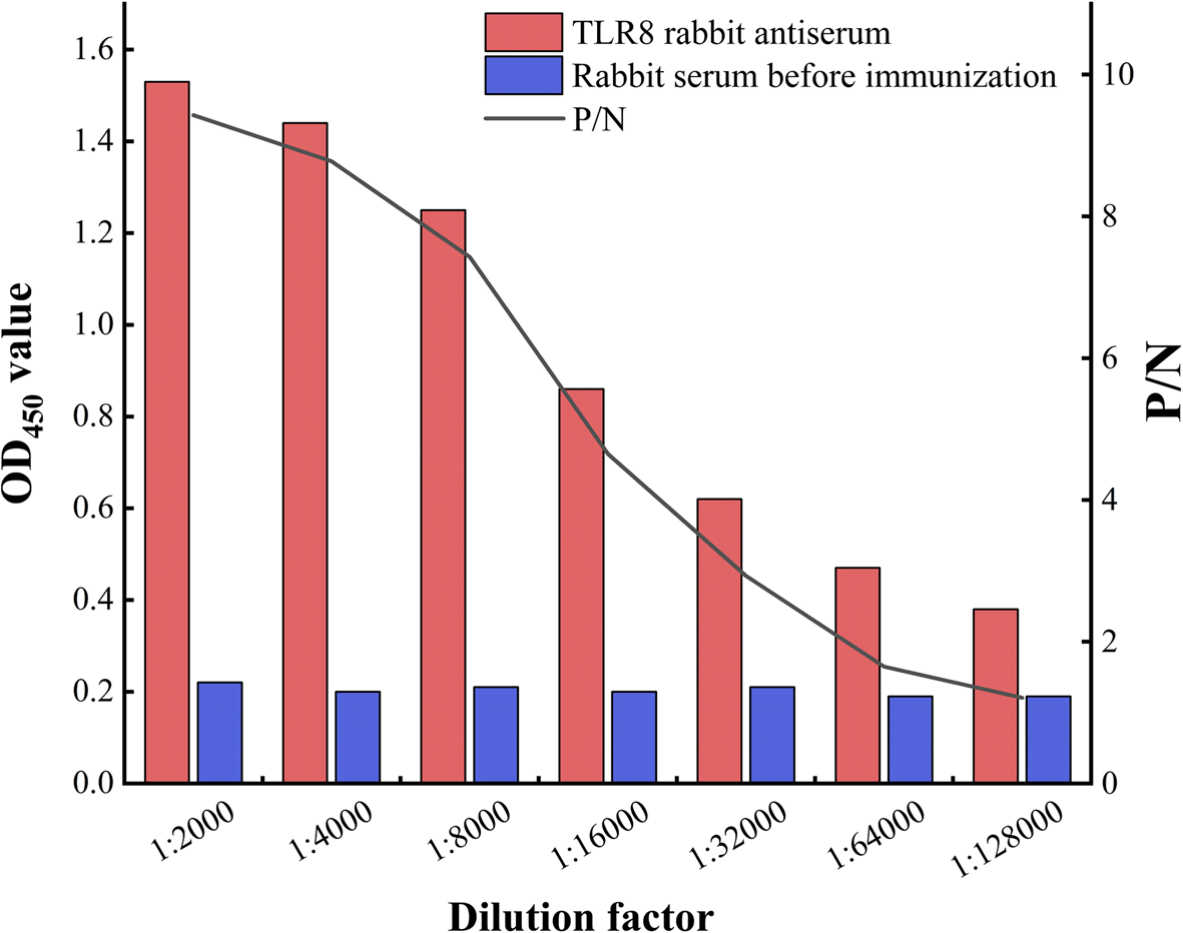
The serum titer detection of rabbit anti-Bactrian camel TLR8 polyclonal antibody

The results of indirect ELISA demonstrated (Fig. 5) that when the rabbit antiserum and pre-immune negative serum were diluted at 1:32 000 and 1:2000, respectively, the OD_450_ (positive)/OD_450_ (negative) value exceeded 2.1. This indicated that the titer of TLR8 polyclonal antibody reached 1:32 000, underscoring the good immunogenicity of TLR8 recombinant protein in inducing specific antibody production in rabbits.

**Fig. 6:**
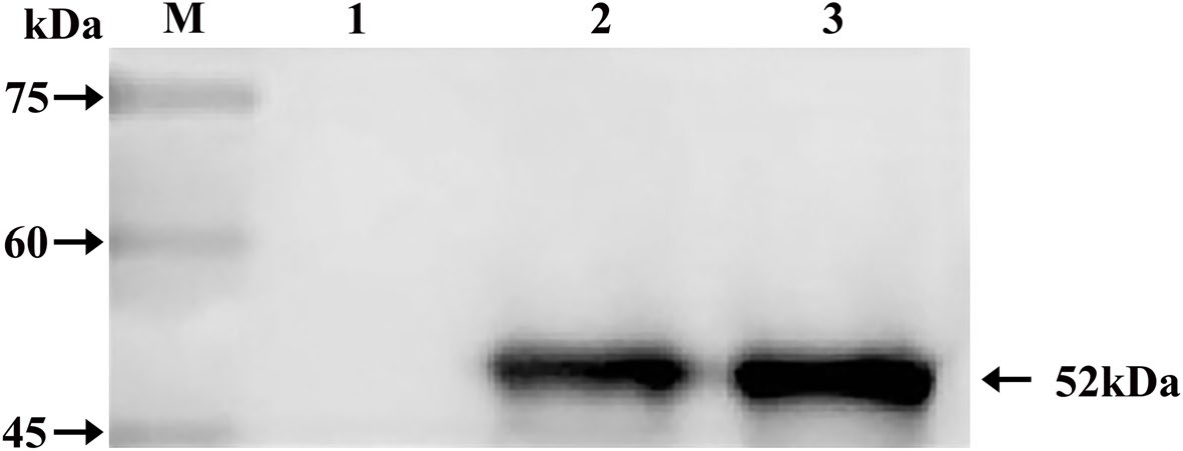
Western blotting results of the TLR8 polyclonal antibody in Bactrian camels. **M** Protein molecular weight Marker; **1** Before induction of the recombinant protein; **2-3** After induction of the recombinant protein. The gel strip of an appropriate size was cut at the 52 kDa, and then electrotransferred to a polyvinylidene fluoride (PVDF) membrane. A cropped image is provided, not a full-length image

To evaluate the specificity of the TLR8 polyclonal antibody, Western blotting was conducted. The results (Fig. 6) revealed a distinct band between 45 and 60 kDa on the PVDF membrane, consistent with the expected size of 52 kDa. This single band indicated that the polyclonal antibody prepared in this study exhibited good reactivity and specific binding to the TLR8 recombinant protein.

### Distribution characteristics of TLR8 in the spleens of Bactrian camels of different ages

**Fig. 7:**
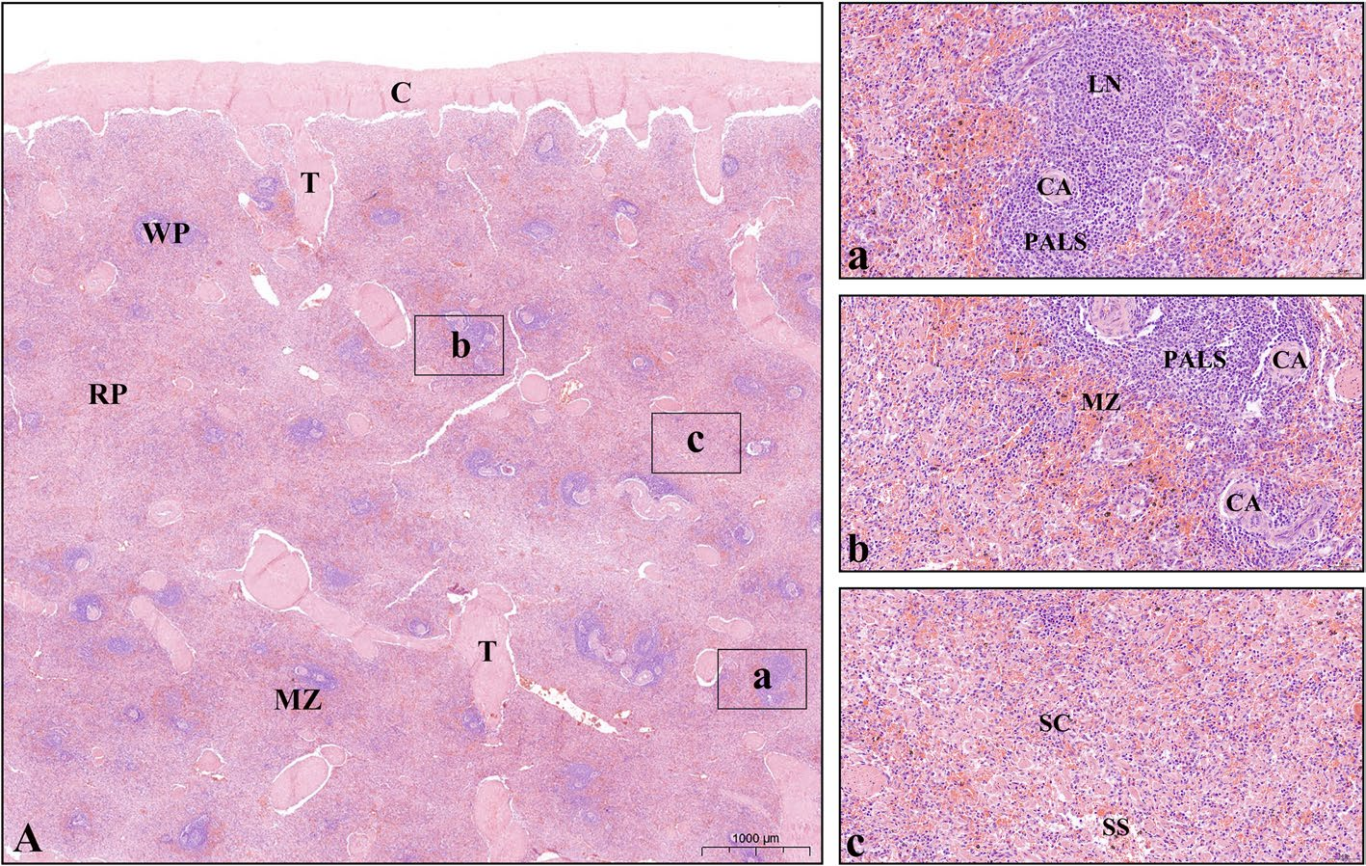
Histological characteristics of the spleens of Bactrian camels. **A** Histological structures of the spleens of Bactrian camels. Bactrian camel spleens are composed of surface capsules (**C**), deep parenchyma, and interstitium. The capsule extends into the parenchyma to form trabeculae (**T**). In the white pulp (**WP**), there are typical lymphoid nodules (**LN**) and periarterial lymphatic sheaths (**PALS**) surrounding the central artery (**CA**). The marginal zone (**MZ**) is located at the junction of the white pulp and red pulp, where lymphocytes are sparser than in the white pulp and a larger number of erythrocytes can be seen. The red pulp (**RP**) is mainly distributed under the capsule, around the trabeculae and outside the marginal zone. It is composed of the splenic cord (**SC**) and splenic sinus (**SS**). **a-c** The partial enlarged images of the white pulp, marginal zone and red pulp of the spleen, respectively. The microscopic magnification of (**A**) is 10×, and (**a-c**) is 200×

**Fig. 8:**
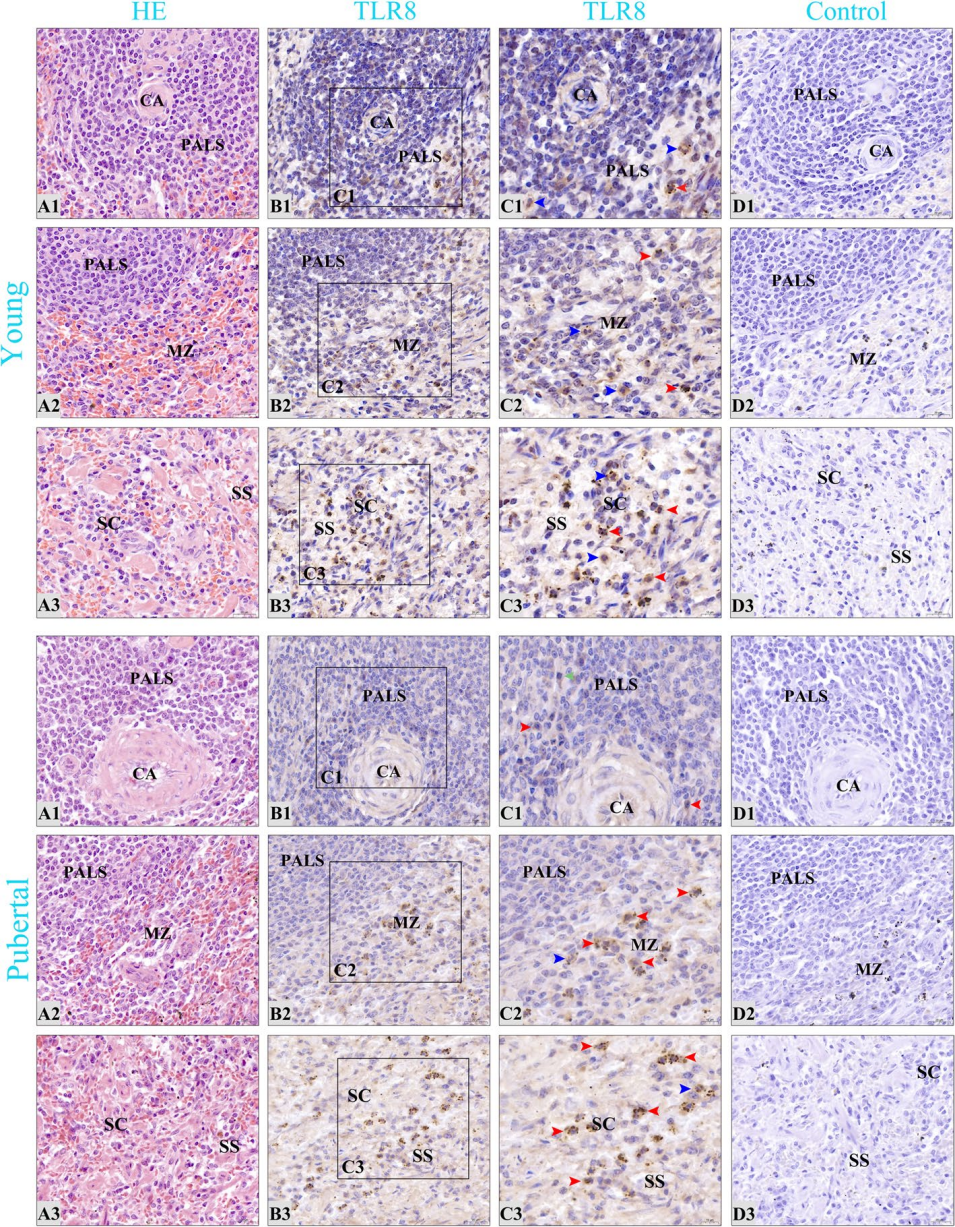
Distribution characteristics of TLR8 positive cells in the spleens of young and pubertal camels. **A1-A3** The white pulp, marginal zone, and red pulp of spleens, respectively. **CA** central artery; **PALS** periarterial lymphatic sheaths; **MZ** marginal zone; **SC** splenic cord; **SS** splenic sinus. From left to right in each column, the sections are stained with H&E, immunohistochemistry for TLR8, and hematoxylin counterstain (as negative control), respectively. **C1-C3** Partial enlarged view of the corresponding sections in **B1-B3**; macrophages are marked by red arrows, neutrophils by blue arrows, and dendritic cells by green arrows; microscopic magnification is 1000×. Except for **C1-C3**, all other magnifications are 400×

**Fig. 9:**
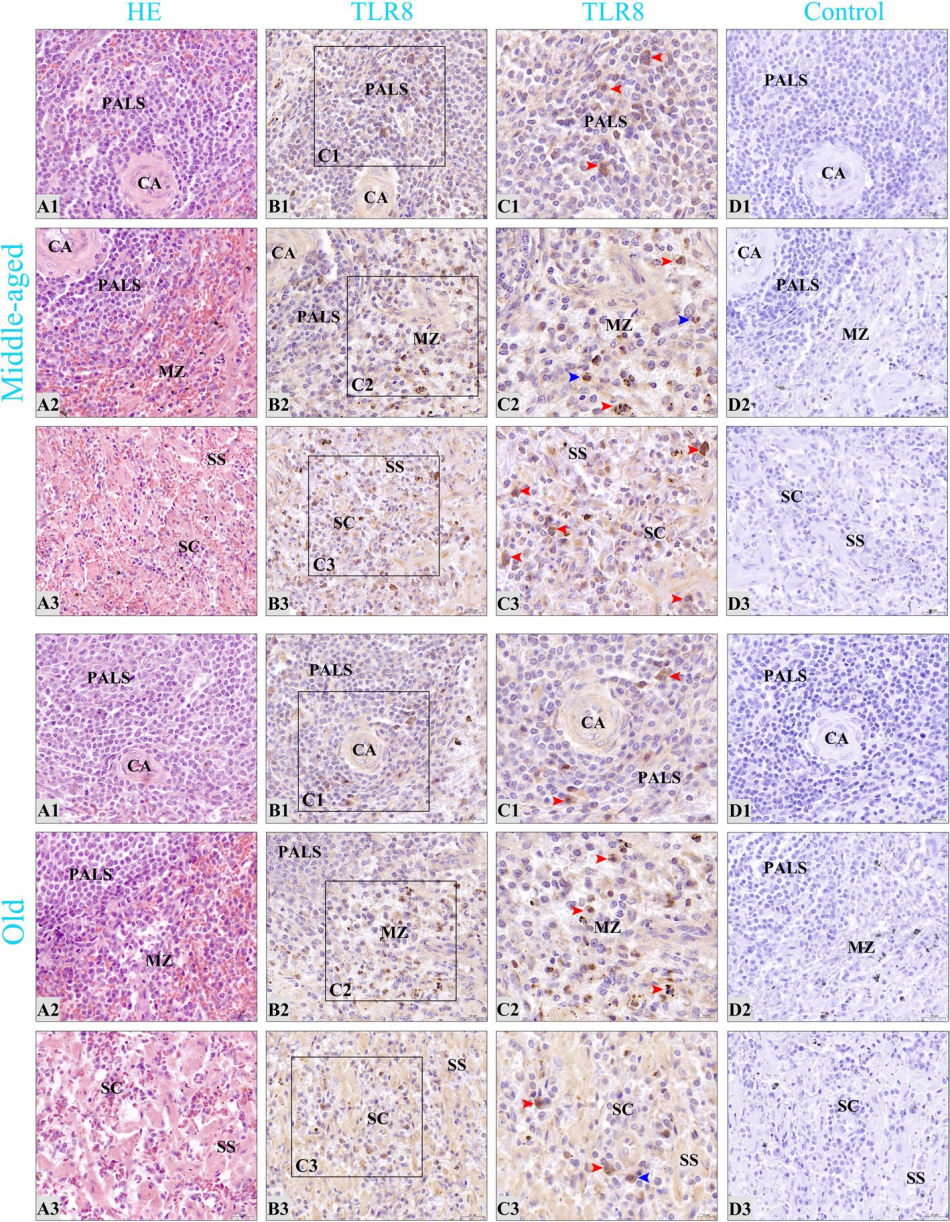
Distribution characteristics of TLR8 positive cells in the spleens of middle-aged and old camels. **A1-A3** The white pulp, marginal zone, and red pulp of spleens, respectively. **CA** central artery; **PALS** periarterial lymphatic sheaths; **MZ** marginal zone; **SC** splenic cord; **SS** splenic sinus. From left to right in each column, the sections are stained with H&E, immunohistochemistry for TLR8, and hematoxylin counterstain (as negative control), respectively. **C1-C3** Partial enlarged view of the corresponding sections in **B1-B3**; macrophages are marked by red arrows, neutrophils by blue arrows, and microscopic magnification is 1000×. Except for **C1-C3**, all other magnifications are 400×

Histologically, the spleens of Bactrian camels (Fig. 7) were categorized into three regions: the white pulp, marginal zone, and red pulp. Immunohistochemical staining results (Figs. 8 and 9) revealed that TLR8 positive cells exhibited a round or oval shape with abundant cytoplasm, large size, and relatively small nuclei, characteristic of macrophages. Some positive cells exhibited rod-shaped or clearly lobulated nuclei, while a small fraction of positive cells had dendritic features with multiple pseudopodial protrusions, suggesting the presence of TLR8 expression in neutrophils and dendritic cells.

In pubertal camels, TLR8 positive cells were scattered in the periarterial lymphatic sheath of the white pulp (Fig. 8B1), primarily comprising macrophages, followed by neutrophils, and relatively fewer dendritic cells. The positive cells were diffusedly distributed in the marginal zone (Fig. 8B2), primarily macrophages, followed by neutrophils. A diffuse distribution pattern was also observed in the splenic sinus and splenic cord of the red pulp (Fig. 8B3), predominantly comprising macrophages, followed by neutrophils. The distribution pattern of positive cells in the spleen of young (Fig. 8B1-B3), middle-aged (Fig. 9B1-B3) and old (Fig. 9B1-B3) camels was largely similar to that of pubertal camels, with a predominant presence in the red pulp and marginal zone, and relatively fewer positive cells in the white pulp.

### Changes in the distribution density of TLR8 positive cells with age

**Fig. 10:**
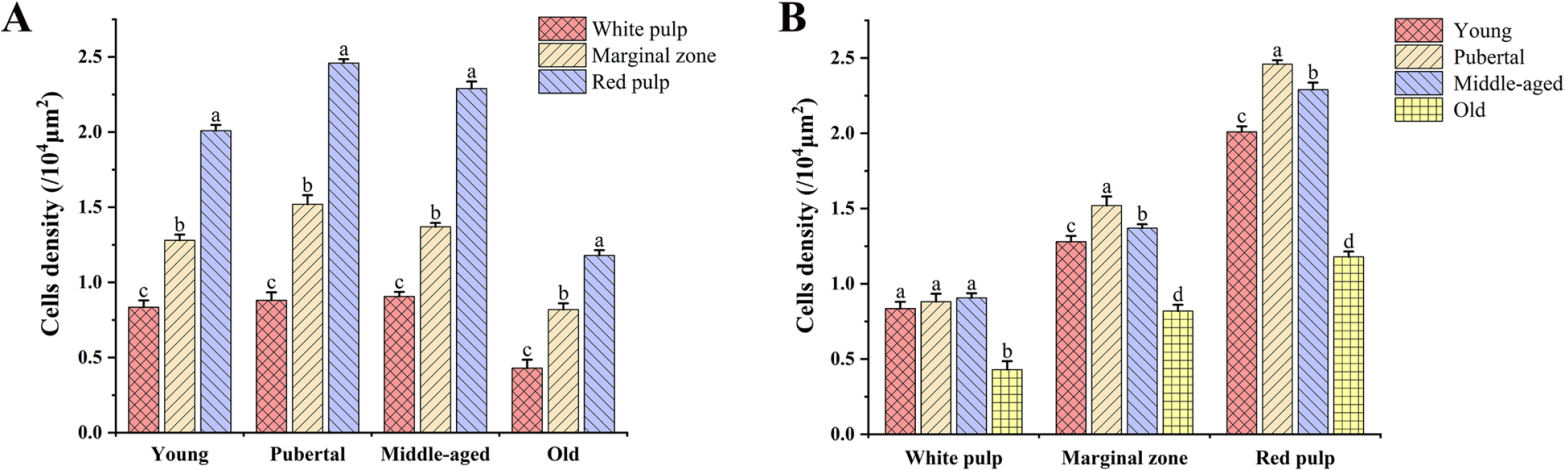
Distribution density of TLR8 positive cells in the spleens of Bactrian camels of different ages. **A **Distribution density of TLR8 positive cells in different spleen regions at the same age;** B **Distribution density of TLR8 positive cells in the same spleen region at different ages; Columns with different lowercase letters represent significant differences (*P*<0.05), while identical lowercase letters represent no significant differences (*P*>0.05)

**Fig. 11:**
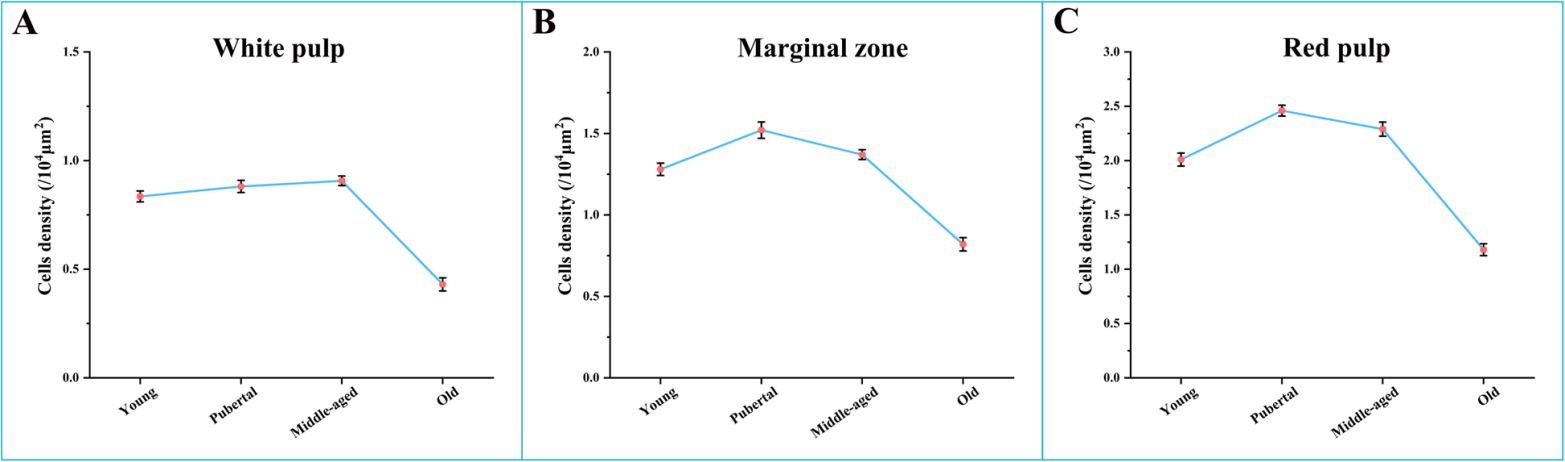
Age-related changes of TLR8 positive cells density in different spleen regions. **A, B and C** indicate the age-related changes of the distribution density of TLR8 positive cells in the white pulp, marginal zone and red pulp, respectively. The distribution density of positive cells in the marginal zone and the red pulp from high to low was pubertal, middle-aged, young and old camels, and there were significant differences among different age groups (*P*<0.05). The overall trend was first an increased and then a decreased in density. The distribution density in the white pulp was not significantly different among young, pubertal, and middle-aged camels (*P*>0.05), but was significantly decreased in old camels (*P*<0.05)

Statistical analysis of the distribution density of TLR8 positive cells in different spleen regions of Bactrian camels (young, pubertal, middle-aged, and old) indicated (Fig. 10A) that the distribution density of positive cells, from highest to lowest, in different regions of the spleen was observed in the red pulp, marginal zone, and white pulp, with significant differences between these regions (*P*<0.05). Specifically, the distribution density of positive cells in the red pulp was significantly higher than in the marginal zone and white pulp (*P*<0.05), while the distribution density in the marginal zone was significantly higher than in the white pulp (*P*<0.05). Furthermore, comparing the distribution density of positive cells in the same spleen region among different age groups revealed that the distribution density in the red pulp, from highest to lowest, occurred in pubertal, middle-aged, young, and old camels, with significant differences among age groups (*P*<0.05). Overall, the trend indicated an initial increase followed by a decrease (Figs. 10B, 11C), with a similar pattern observed in the marginal zone (Figs. 10B, 11B). Remarkably, the distribution density of positive cells in the white pulp did not significantly differ among young, pubertal, and middle-aged camels (*P*>0.05) but was notably reduced in old camels (*P*<0.05) (Figs. 10B, 11A).

## Discussion

Prokaryotic expression represents a widely employed method for obtaining protein antigens due to its numerous advantages, including a clear genetic background, cost-effectiveness, rapidity, simplicity of operation, and the ability to meet the rapid antigen production requirements for antibody development [36, 37]. Throughout the process of prokaryotic expression, the expression level of the target protein is not only influenced by factors such as bacterial strain type and expression plasmid, but also related to other factors, including inducer concentration, induction time and induction temperature [38]. In this study, it was observed that under the optimal IPTG concentration, the expression of the TLR8 recombinant protein exhibited a pattern of initially increasing and subsequently decreasing with prolonged induction time. Notably, the recombinant protein was primarily expressed in the form of inclusion bodies. These findings align with previous studies involving the preparation of polyclonal antibodies via prokaryotic expression [30, 39]. This observed pattern can be attributed to several factors. Firstly, bacterial populations undergo distinct growth phases, including lag phase, logarithmic phase, stationary phase, and decline phase [40]. The initial increase in the expression level of the recombinant protein is likely associated with the rapid proliferation of the bacterial population in a stable geometric progression following the transition from the lag phase to the logarithmic phase [41]. As the number of viable bacteria increases linearly, the expression of the target protein also rises. However, as the induction time progresses, the bacterial population transitions from the stationary phase to the decline phase. During this phase, there is a substantial increase in the number of dead bacteria, bacterial cells age, cell walls thicken [41], and incomplete protein release occurs during ultrasonic fragmentation, leading to a reduction in the content of the recombinant protein. The presence of the recombinant protein in the form of inclusion bodies in the precipitate can be attributed to the high expression of the protein in *Escherichia coli *[42], and codon optimization of the truncated fragment may contribute to the rapid expression of the recombinant protein and the formation of inclusion bodies. Additionally, the low hydrophilicity of the TLR8 recombinant protein may be another significant factor for its expression in the form of inclusion bodies [30]. TLR8 primarily recognizes PAMPs through its extracellular domain, so the extracellular domain of TLR8 was selected for prokaryotic expression, and the expected-sized recombinant protein was obtained. Subsequently, the TLR8 polyclonal antibody was successfully prepared by immunizing rabbits with the recombinant protein. The antibody exhibited a titer of 1:32,000, as determined by indirect ELISA. Western blotting confirmed the presence of the target band at 52 kDa, consistent with the expected size. These results indicating that this study successfully prepared a highly efficient and specific rabbit anti-Bactrian camel TLR8 polyclonal antibody.

Innate immunity is a series of defense mechanisms formed during the germline evolution of the body, and pattern recognition receptors (PRRs) play an important role in innate immune responses [43]. TLR8, a crucial PRR, plays a pivotal role in recognizing specific PAMPs, thereby activating the body's innate immune response. Subsequently, this activation induces adaptive immune responses to clear the infecting pathogens [44]. The spleen, as the largest peripheral immune organ in the body, harbors a diverse population of immune-active cells and immune-related molecules. It serves as a crucial response site for both innate and adaptive immunity, and has a wide range of immunological functions [45]. Importantly, the spleen exhibits high expression levels of TLR8 [46], and the multiple immunological effects mediated by TLR8 are integral to its immune function. Consequently, this study sought to investigate the distribution patterns of TLR8 in the spleen and examine how these patterns change with age. The results of immunohistochemical staining consistently revealed that TLR8 positive cells displayed similar distribution patterns in the spleens of Bactrian camels across different age groups. These positive cells were primarily scattered throughout three key regions: the periarterial lymphatic sheath (PALS) of the white pulp, the marginal zone, and the splenic cord and splenic sinus of the red pulp. These positive cells were primarily identified as macrophages but were also detected in neutrophils and dendritic cells. These findings were basically consistent with the previous studies that reported the distribution of TLR1 and TLR2 positive cells in the spleen and lymph nodes [47, 48], and were also consistent with the cellular expression profile of TLR8 [9, 10]. This suggests that macrophages may be the primary cell type through which TLR8 exerts its immune functions within the spleen. The white pulp of the spleen serves as the primary immune region, housing the PALS, which contains densely packed T lymphocytes and is a key site for cellular immune responses [49, 50]. The presence of scattered TLR8 positive cells within the PALS suggests their potential role in efficiently capturing antigens and presenting them to T lymphocytes. The marginal zone, as the region where arterial blood first flows through, is the site where the spleen first contacts antigens and induces immune response [49, 50]. The presence of scattered TLR8 positive cells within the marginal zone may facilitate the effective clearance of foreign substances and antigens from the bloodstream. The red pulp constitutes the primary blood-filtering region of the spleen [49]. TLR8 positive cells scattered within the splenic cord and splenic sinus likely contribute to the phagocytosis and clearance of blood-borne antigens, as well as aged, malformed, and dysfunctional red blood cells. This process aids in maintaining iron homeostasis within the body. Macrophages, neutrophils, and dendritic cells in the spleen are capable of recognizing specific PAMPs through TLR8 homodimers, thereby mediating innate immune responses. The widespread distribution of TLR8 positive cells across the various regions of the spleen appears to be essential for the capture, uptake, presentation, and clearance of antigens. Importantly, this distribution pattern remains consistent across different age groups, suggesting that age does not significantly alter these fundamental immunological characteristics within the spleen.

The statistical analysis of our findings revealed a distinct hierarchy in the distribution density of TLR8 positive cells among different regions of the spleen, regardless of age. Specifically, this hierarchy ranked the red pulp, marginal zone, and white pulp in descending order of distribution density, with statistically significant differences observed between these regions (*P*<0.05). These results suggest that both the red pulp and marginal zone are key players in the splenic innate immune response. This distribution pattern can be attributed to the fact that approximately two-thirds of splenic tissue comprises the red pulp, with the splenic sinuses and splenic cords being the primary sites for the distribution of innate immune cells [51]. Notably, TLR8 positive cells are predominantly macrophages, neutrophils, and dendritic cells, which explains the higher density of TLR8 positive cells in the red pulp. Furthermore, the red pulp mainly serves as a blood filter, performing the functions of hematopoiesis, blood storage and removal of foreign materials [49, 50]. Innate immune cells are predominantly situated in the red pulp [51], enabling them to efficiently phagocytose aging and deceased red blood cells, detect pathogens, and respond to tissue damage. The marginal zone serves as the anatomical site where blood-borne pathogens enter the white pulp from the red pulp bloodstream [49, 50]. Given the higher distribution of TLR8 positive cells in the marginal zone, it is likely that these positive cells play a crucial role in capturing blood-borne antigens and apoptotic cells. This localization is integral to immune surveillance and adaptive immune responses. In contrast, the white pulp constitutes the primary region for cellular and humoral immune responses within the spleen, making up one-fifth of the splenic tissue [45, 49]. It primarily consists of T and B lymphocytes, with fewer innate immune cells [51]. Scattered TLR8 positive cells within the white pulp can effectively mediate antigen presentation and regulate T cell responses. Moreover, our analysis of the distribution density of TLR8 positive cells in the marginal zone and red pulp revealed similar age-related trends. These trends were characterized by an initial increase followed by a subsequent decrease from youth to old age. These findings align with previous studies reporting on the distribution of secretory immunoglobulin A (SIgA) and IgG antibody-secreting cells within the small intestine and aggregated lymphoid nodule areas of Bactrian camels across different age groups [52, 53]. As Bactrian camels mature, their various growth indicators and organ functions gradually improve, and the immune function of the spleen also reaches the strongest. In this developmental process, the increased presence of TLR8 positive cells, such as macrophages, neutrophils and dendritic cells, likely enhances the body's ability to resist microbial invasions. Conversely, as camels age, the density of TLR8 positive cells within each spleen region significantly decreases (*P*<0.05). Several studies have reported age-related reductions in the numbers of splenic macrophages and granulocytes [54], as well as decreased expression and dysfunction of TLRs in macrophages and neutrophils [55–57]. Additionally, aging is associated with a decline in the number of splenic dendritic cells [58] and a decrease in macrophage capacity to phagocyte apoptotic cells [57]. Thus, it can be inferred that the effect of aging on the distribution density of TLR8 positive cells within each spleen region may be mediated by reductions in both the number of positive cells and the expression of TLR8 within the corresponding immune cells. These findings bolster the basis for further research into immunomorphology and immunosenescence within the spleens of Bactrian camels.

## Conclusions

This study successfully achieved several critical objectives. Firstly, we effectively constructed the pET-28a-TLR8 recombinant plasmid, which constitutes a fundamental step in our research. Secondly, we prepared a rabbit anti-Bactrian camel TLR8 polyclonal antibody characterized by its good specificity. Finally, we conducted a comprehensive investigation into the distribution patterns and age-related changes of TLR8 within the spleens of Bactrian camels. Our results unveiled that TLR8 positive cells exhibit a predominant presence in the splenic red pulp and marginal zone. This observation underscores the vital role played by these two regions in orchestrating the splenic innate immune response. Notably, we observed that aging exerts a significant influence on the density of TLR8 positive cells within these regions, leading to a decrease in density. Remarkably, this age-related effect did not alter the dispersed distribution patterns of TLR8 positive cells. These findings contribute valuable insights that will undoubtedly support further inquiries into the immunomorphology and immunosenescence of the spleen in Bactrian camels. Moreover, they offer a promising avenue for exploring the intricate mechanisms governing the TLR8 signaling pathway.

## Materials and methods

### Experimental animals and tissues collection

A total of twelve healthy Alashan Bactrian camels were categorized into four age groups: young (1–2 years, *n*=3), pubertal (3–5 years, *n*=3), middle-aged (6–16 years, *n*=3), and old (17–20 years, *n*=3). These camels were sourced from a local slaughterhouse in Minqin County, Gansu Province, China. Prior to slaughter, these camels were not starved and were humanely anesthetized intravenously using sodium pentobarbital (20 mg/kg) to minimize any potential discomfort. Subsequently, exsanguination was performed to ensure euthanasia. The spleen was meticulously isolated from the dorsal aspect of the rumen in each Bactrian camel. Following collection, the spleen tissues were gently rinsed with PBS to eliminate surface blood. The collected tissues were preserved in appropriately labeled sampling bottles with 4% paraformaldehyde (Solarbio, Beijing, China) for the subsequent preparation of paraffin sections.

### Synthesis of TLR8 gene and construction of recombinant plasmid in Bactrian camel

The TLR8 mRNA sequence of the Bactrian camel (GenBank: XM_010966216.2) spans 4729 bp, with a coding sequence of 3237 bp (position 247-3483), encoding a total of 1078 amino acids. The transmembrane structure of the TLR8 protein was predicted using TMHMM Server 2.0 software (https://services.healthtech.dtu.dk/services/TMHMM-2.0/), and the extramembrane region was intercepted using Editseq program of DNAStar7.0. SignalP-5.0 Server software (https://services.healthtech.dtu.dk/services/SignalP-5.0/) was used to predict and cut off the signal peptide of the TLR8 protein. Subsequently, the hydrophilicity and antigenic epitope of the TLR8 protein were predicted using Protean program of DNASTAR7.0, and the extramembrane amino acid sequence with a higher antigenic index was selected as the target fragment. Primer Premier 5.0 was utilized to forecast the restriction sites in the base sequence corresponding to the amino acid sequence. Upstream of the base sequence, the* BamH* I restriction site and the start codon were appended, while downstream, the *Xho* I restriction site and the stop codon were added. Finally, the intercepted TLR8 base sequence was optimized, synthesized (Jinweizhi Biotechnology Co., Ltd.), connected with pET-28a (+) vector (Solarbio, Beijing, China), and transformed into DH5α competent cells (Solarbio, Beijing, China) for culture. The plasmid was extracted for sequencing and the positive recombinant plasmid with correct sequencing was named pET-28a-TLR8.

### Prokaryotic expression of pET-28a-TLR8 recombinant plasmid

The pET-28a-TLR8 recombinant plasmid was introduced into BL21 competent cells (C1400, Solarbio, Beijing, China), and 700 µL LB liquid medium without Kanamycin (Kan^+^) was added for culture. Then, 100 µL of bacterial liquid was plated on LB solid medium containing Kan^+^ and incubated at 37 °C overnight. A single colony was selected, inoculated in 5 mL LB liquid medium containing Kan^+^, and cultured at 37°C until the OD_600_ value reached 0.6-0.8. Before induction, 1 mL of bacterial liquid was withdrawn as a control, and the remaining bacterial culture was induced using 1.0 mmol/L IPTG for 6 h. Finally, all samples were detected by 12% sodium dodecyl sulfate polyacrylamide gel electrophoresis (SDS-PAGE).

### Optimization of expression conditions of TLR8 recombinant protein in Bactrian camel

To enhance the expression level of TLR8 recombinant protein, optimization of IPTG concentration and induction time was performed. Different IPTG concentrations (0.1 mmol/L , 0.3 mmol/L, 0.5 mmol/L, 0.7 mmol/L, 0.9 mmol/L, 1.0 mmol/L) were used to induce the expression of TLR8 recombinant protein, and all samples were detected by 12% SDS-PAGE. The impact of varying IPTG concentrations on recombinant protein expression was assessed to identify the optimal inducer concentration. To determine the optimal induction time, under the optimal IPTG concentration, the expression level of TLR8 recombinant protein was detected by 12% SDS-PAGE at different induction times (2 h, 4 h, 6 h, 8 h, 10 h).

### Purification of TLR8 recombinant protein in Bactrian camel

The expression of TLR8 recombinant protein was induced at the optimal IPTG concentration and duration. A control sample of 1 mL bacterial liquid was collected before ultrasonication, while the remainder was disrupted by ultrasound until the liquid became clear. After centrifugation, the lysed supernatant and precipitate were collected respectively for 12% SDS-PAGE detection to determine the expression form of the recombinant protein.

Based on the results obtained from 12% SDS-PAGE detection, lysed precipitates from recombinant bacteria were collected for protein purification. The target protein was purified from the lysed precipitates using the His Tag Protein Purification Kit (Inclusion Body Protein) (CW0893S, CWBIO, Jiangsu, China), following the manufacturer’s instructions. The efficiency of protein purification was evaluated using 12% SDS-PAGE, and the protein content was determined using an Ultraviolet spectrophotometer (GeneQuant 1300, General Electric Co., USA).

### Preparation of rabbit anti-Bactrian camel TLR8 polyclonal antibody

To prepare a rabbit anti-Bactrian camel TLR8 polyclonal antibody with good specificity and high titer,  we established a well-planned immunization protocol. Three healthy adult New Zealand white rabbits (male, 2.7 ± 0.3 kg) were procured from the Experimental Animal Center at Lanzhou Veterinary Research Institute, Chinese Academy of Agricultural Sciences. After all rabbits were fed adaptively for 7 days, a small volume of blood was collected from the ear veins before immunization. The serum obtained from this blood collection served as a negative control and was stored at -80 ℃ for future use. The first immunization involved emulsifying the purified TLR8 recombinant protein with an equal volume of Freund’s complete adjuvant (F5881, Sigma, Missouri, USA). Subsequently, the rabbits were immunized by subcutaneous multi-point injections at the back, scapular and popliteal lymph nodesa at dose of 800 µg per rabbit. One week later, a second immunization was administered. In this case, the purified protein was emulsified with an equal volume of Freund’s incomplete adjuvant (F5506, Sigma, Missouri, USA), and then the rabbits were immunized by subcutaneous multi-point injections at the back and popliteal lymph nodes at a dose of 400 µg per rabbit. Subsequent immunizations were conducted every other week, with the same immunization method and dose as the second round. One week following the fourth immunization, the rabbits were anesthetized by injecting sodium pentobarbital (30 mg/kg) into the ear vein. Blood was then collected from the heart to ensure euthanasia, with meticulous attention to minimizing animal suffering. The collected blood was centrifuged to obtain rabbit anti-Bactrian camel TLR8 poly-antiserum, which was subsequently stored at -80°C.

### Detection of serum titer of Bactrian camel TLR8 polyclonal antibody

The antibody titer was determined by an indirect enzyme-linked immunosorbent assay (ELISA) method, with the following key steps: 1) The purified TLR8 recombinant protein, used as the antigen, was added to the ELISA plate at a concentration of 5μg/well, and kept at 4 ℃ overnight. 2) The wells were washed three times for 5 min with TBST, followed by blocking with 5% skim milk powder (100 µL/well) at 37 ℃ for 1 h. 3) After another round of washing with TBST, the rabbit antiserum was added at varying dilutions (ranging from 1:2000 to 1:128000) at 100 µL/well, and the plates were incubated at 37 ℃ for 1 h. 4) Subsequent to another round of washing with TBST, HRP-labeled goat anti-rabbit IgG (BA1054, BOSTER, Wuhan, China), diluted to 1:8000, was added at 100 µL/well and incubated at 37 ℃ for 1 h. 5) Following another round of washing with TBST, TMB substrate chromogenic solution (PR1200, Solarbio, Beijing, China) was added at 100 µL/well in a dark environment. 6) After color development at room temperature for 15 min, 2 mol/LH_2_SO_4_was added to stop the reaction at 50 µL/well. 7) The absorbance at 450 nm was measured using a ReadMax 1200 microplate reader (Shanghai Shanpu Biotechnology Co., Ltd., Shanghai, China). 8) The highest dilution ratio where OD_positive_/OD_negative_ ≥ 2.1 was determined as the serum titer of the polyclonal antibody. Notably, the antiserum of each gradient was added in parallel three times, and the pre-immune rabbit serum, diluted 1:2000, served as the negative control. All other procedures were consistent with those of the experimental group, and a blank control was also set.

### Western blotting identification of Bactrian camel TLR8 polyclonal antibody

The purified TLR8 recombinant protein was subjected to electrophoresis on a 12% SDS-PAGE gel. Subsequently, a gel strip of appropriate size, corresponding to 52 kDa, was cut and electrotransferred onto a polyvinylidene fluoride (PVDF) membrane. The membrane was then blocked with TBST containing 5% skim milk powder at 37 ℃ for 2 h. Following blocking, the membrane was incubated with rabbit antiserum (diluted 1:800) overnight at 4 ℃. After washing with TBST three times for 15 min each, the membrane was incubated with HRP-labeled goat anti-rabbit IgG (diluted 1: 8000) at room temperature for 2 h. Finally, after washing again, ECL luminescent solution (PE0010, Solarbio, Beijing, China) was added for color development, and the membrane was exposed and photographed.

### Hematoxylin-eosin (H&E) and immunohistochemistry (IHC) staining

The fixed spleen tissues were respectively embedded in paraffin, and then cut into 5-µm-thick sections using a microtome (HistoCore MULTICUT, Leica, Germany) for routine hematoxylin and eosin (H&E) staining. The histological structure of the Bactrian camel spleen was carefully observed under a light microscope, and images were captured using the Olympus DP-71 microscopy system (Olympus, Hamburg, Germany).

The prepared paraffin sections were performed to indirect SABC immunohistochemical staining, with the following primary steps: 1) The sections were deparaffinized, dehydrated and washed. 2) 3% H_2_O_2_ was added and incubated for 15 min at room temperature to eliminate endogenous peroxidase activity, and then washed with distilled water three times for 5 min each. 3) 0.1% trypsin was added and incubated for 40 min at 37 ˚C to repair the antigen, and then washed with distilled water three times each for 5 min. 4) To avoid non-specific binding, the sections were blocked with 5% BSA and incubated for 45 minutes at 37°C without washing. 5) The rabbit antiserum (diluted 1: 900), serving as the primary antibody, was added to the positive group and incubated at 4 ˚C overnight to allow the antigen and antibody to react fully. In the negative control group, PBS solution was used in place of the primary antibody. In subsequent experiment, both were washed separately with PBS five times for 5 min each. 6) Biotin-labeled goat anti-rabbit IgG from an immunohistochemical kit (SA1022, BOSTER, Wuhan, China), as the secondary antibody, was added and incubated for 1 h at 37˚C, and then washed with PBS five times for 5 min each. 7) SABC was added and incubated for 30 min at 37˚C, and then washed with PBS five times for 5 min each. 8) DAB colour rendering was performed at room temperature in a dark environment. Subsequently, the sections were counterstained with hematoxylin dyeing solution (Solarbio, Beijing, China) and sealed with neutral balsam (Solarbio, Beijing, China).

### Statistical analysis

Five sections were randomly chosen from each sample, and ten microscopic fields were randomly selected from each of the white pulp, marginal zone, and red pulp in each section for observation and photomicrography. TLR8 positive cell counts were performed in each microscopic field, and the density of positive cells was calculated using Image-Pro Plus 6.0, respectively. Statistical analysis was conducted to assess significant differences in the distribution density of TLR8 positive cells within different regions of the spleen within the same age group, as well as within the same region of the spleen among different age groups. This analysis was carried out using one-way analysis of variance (ANOVA) followed by Duncan’s multiple range test. The data analyses were performed using IBM SPSS V.23.0 (SPSS Inc., Chicago, USA), and the graphs were constructed using OriginPro 2018C software (OriginLab Inc., Northampton, USA). Significance was established at *P*<0.05.

### Supplementary Information

**Additional file 1. **

## Data Availability

The data that support the findings of this study are available from the article or supplementary material, further inquiries can be directed to the corresponding authors.

## Abbreviations

TLRs: Toll-like receptors
TLR8: Toll-like receptor 8
PAMPs: Pathogen-associated molecular patterns
LRR: Leucine-rich repeat
TIR: Toll/IL-1 receptor
MyD88: Myeloid differentiation factor 88
IRAK1: IL-1R-associated kinase 1
TRAF6: TNF receptor-associated factor 6
IRF7: Interferon regulatory factor 7
SIgA: Secretory immunoglobulin A
