# Supplementary figures and images for: Antibody preparation and age-dependent distribution of TLR8 in Bactrian camel spleens
Source: BMC Vet Res. 2023 Dec 16;19:276. doi: 10.1186/s12917-023-03812-z (PMC10725000; doi:10.1186/s12917-023-03812-z)

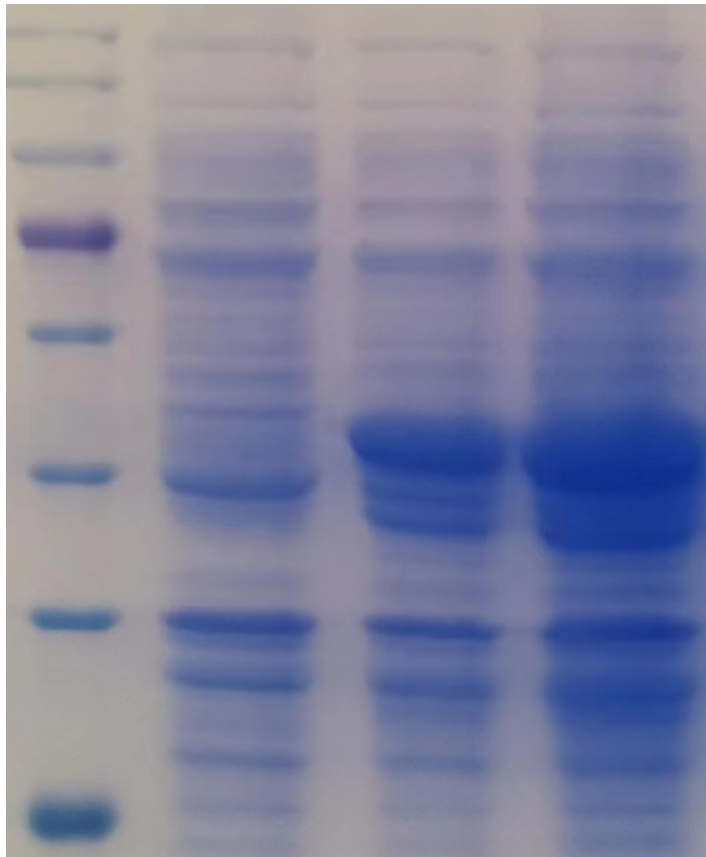

(Figure A)

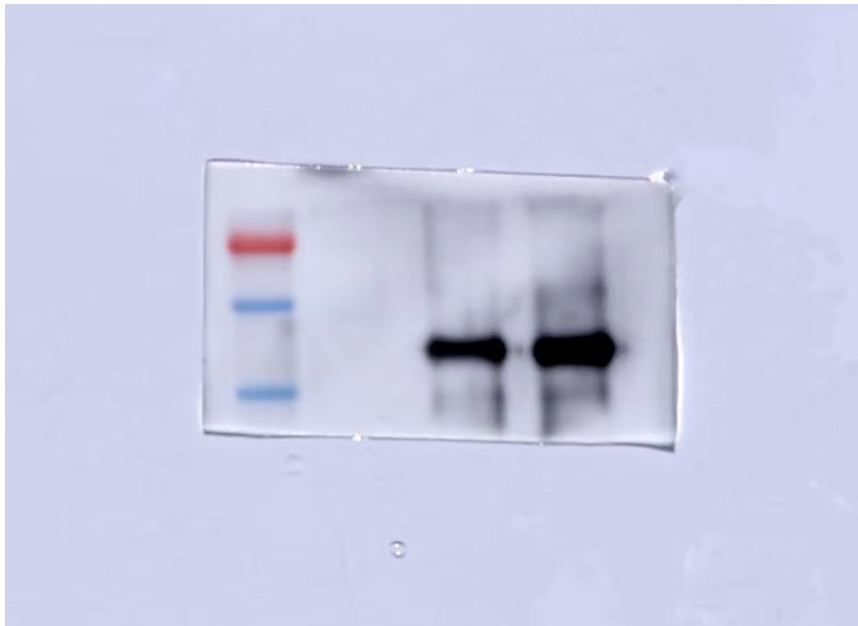

(Figure B)

Supplement: Supplementary file 1 — Additional file 1. [file 12917_2023_3812_MOESM1_ESM.pdf]
